# A New Medical Journal

**Published:** 1845-06

**Authors:** 


### A NEW MEDICAL JOURNAL.

We have received the first number of a monthly journal of medicine, issued at St. Louis, by the Medical Faculty of Kemper College. Among other papers, this number contains communications from Professors Barbour and McDowell on practical subjects, and evinces a full share of spirit. The medical schools of St. Louis have, now, each a journal and organ, and we may expect to see an animated contest between them for the largest amount of public favor. *E collisione scintilla* is an old maxim, the truth of which, we have no doubt, our St. Louis contemporaries will confirm.

